# Bio-Based Waterborne PU for Durable Textile Coatings

**DOI:** 10.3390/polym13234229

**Published:** 2021-12-02

**Authors:** David De Smet, Madeleine Wéry, Willem Uyttendaele, Myriam Vanneste

**Affiliations:** Centexbel, Technologiepark 70, 9052 Zwijnaarde, Belgium; mw@centexbel.be (M.W.); wu@centexbel.be (W.U.); mv@centexbel.be (M.V.)

**Keywords:** polyurethane (PU), coating, bio-based, dispersion, textile

## Abstract

Polyurethane (PU) coatings are often applied on high added value technical textiles. Key factor to success of PU coatings is its versatility and durability. Up to today most PU textile coatings are solvent-based or water-based. Recent advances are made in applying bio-based PU on textiles. Currently, polymers made from renewable raw materials are experiencing a renaissance, owing to the trend to reduce CO_2_ emissions, the switch to CO_2_-neutral renewable products and the depletion of fossil resources. However, the application of bio-based coatings on textiles is limited. The present paper discusses the potential of a bio-based anionic PU dispersion as an environment friendly alternative for petroleum-based PU in textile coating. Coatings were applied on textile via knife over roll. The chemical, thermal and mechanical properties of the bio-based PU coating were characterised via FT-IR, thermogravimetric analysis, differential scanning calorimetry and tensile test. The performance of the coating was studied by evaluating antimicrobial properties, fire retardancy, the resistance to hydrostatic pressure initially and after washing, QUV ageing and hydrolysis test. The developed bio-based PUD coating complied to the fire retardancy test ISO 15025 and exhibited excellent hydrostatic pressure, QUV ageing resistance, hydrolysis resistance, wash fastness at 40 °C.

## 1. Introduction

Polyurethane (PU) is widely used in diverse applications such as coatings, foams, adhesives, sealants and elastomers. PU can also be used for microcapsules. Isosorbide-based PU microcapsules were prepared and applied on polyamide fabrics [1]. Among organic coatings, PU coatings have unique performances and properties including abrasion resistance, toughness, low temperature flexibility and chemical resistance, and are used in a wide range of applications from automobile finishing to textile coatings [2]. The substitution of fossil and solvent-based PU coatings by waterborne bio-based PU coatings has received much attention due to volatile organic compounds emission, regulation (e.g., REACH regulation of dimethyl formamide), depletion of fossil resources and the goal to lower CO_2_ emissions [3,4,5]. Generally, PU are generated from the reaction between a diisocyanate and a polyol in the presence of a catalyst resulting in an isocyanate terminated pre-polymer. In a second step (chain extension) the prepolymer reacts with a chain extender. The chain extender could be a short chain diol, resulting in urethane bonds, or a short chain diamine resulting in urea bonds [6]. Vegetable oils are one of the most promising exploited renewable resources for PU coatings due to their large availability, good toxicity profile and low cost. Different routes are developed to produce bio-based polyols out of vegetable oils for PU synthesis [7]. Fridrihsone et al. performed LCA study of rapeseed oil-based polyol for PU synthesis. Compared to petrochemical polyols bio-based rapeseed oil polyols have a better environmental performance in 8 out of the 18 ReCiPe midpoint impact categories and lower Cumulative Energy Demand [8]. Another interesting bio-based resource is lignin, which is extensively studied as raw material for PU foams and coatings [9].

The properties of water-based PU dispersion (PUD) depend on the type of polyol (functionality, hydroxy value, …), nature of isocyanate, type of chain extender and emulsifier [10]. Fatty acid-based PUD based on dimer technology improved the hydrolytic and storage stability of polyurethane dispersions and showed improvement in different aspects such as adhesion, hydrophobicity and chemical resistance properties [11]. Liang et al. prepared series of bio-based polyols from olive, castor, corn, canola, rice bran, grape seed and linseed oil. An increase of tensile strength, Young’s modulus and *T*_g_ along with the increase of the hydroxy values of the polyols was reported. However, the elongation at break and thermal stability decreased [7]. Waterborne bio-based transparent polyurethane with good chemical resistance ability, UV-aging capability and biodegradability in nature were synthesised. All PU films showed high elongation at break. The results indicated that the synthesised PU could be used as a green coating [12]. Biodegradable microwave-assisted self-healing PUD were synthesised. Three different PU were developed out of poly (ε-caprolactone) diol and bio-based macroglycol with aliphatic and aromatic diisocyanates. The bio-based macroglycol and chain extender were synthesised via the catalyst-based esterification reactions of dimer acid, poly (ethylene glycol), citric acid and glycerol. The resulting PU exhibited high elongation at break [13]. Bio-based PU dispersions with a novel bio-based internal emulsifier, synthesised by reacting glutaric acid and epoxidized soybean oil, were prepared. Solid content of the dispersion was up to 45% and the resulting bio-based PU films exhibited good toughness and thermal stability [14]. Bio-based PUD were modified with cellulose nanocrystals (as renewable reinforcement) and applied on aluminium plates via dip-coating. The addition of cellulose nanocrystals led to higher hardness and elastic recovery, lower plastic deformation, smaller friction coefficient and improvement of wear resistance of the coating [15]. Waterborne bio-based PUD composed of epichlorohydrin modified lignin is reported [16]. Tung oil-based polyol was also assessed in the synthesis of bio-based PU dispersions. It was reported that the resulting PU exhibited good antibacterial activity, particularly against *Escherichia coli* [17]. Octahydro-2,5-pentalenediol (OPD) was synthesised from naturally occurring citric acid and used together with castor oil as the polyol blends to produce bio-based waterborne PUD. Waterborne polyurethane films with tailorable mechanical performance ranging were prepared and characterised. The tensile strength increased with the ratio between OPD and castor oil increasing, whereas their elongation at break decreased from 192 to 12%. A significant increase in the glass transition temperature, transparency and anticorrosive properties was observed for the resulting polyurethane films with increasing the OPD content [18].

Since PU is obtained via reaction between polyols and isocyanates, bio-based PU can also be obtained by implementing bio-based isocyanates. PUD were made of dimer fatty acid diisocyanates and castor oil [19]. The use of ethyl ester L-lysine diisocyanate and ethyl ester l-lysine triisocyanate in PU films was also reported. Compared to isophorone diisocyanate-based PU, the bio-based PU tend to swell in organic solvents [20]. Bio-based PU adhesives for wood applications were developed by modifying cellulose acetate with 1,6-hexamethylene diisocyanate followed by mixing with a variable amount of castor [21]. Haro et al. reported the reaction of Lignin-based diisocyanate with unmodified lignin resulting in thermoset PU coatings [22]. Bio-based pentamethylene diisocyanate (PDI) has a significant bio-based content and is the first example of bio-based diisocyanate which has been commercialised. The trimeric PDI is commercialised under trade name DESMODUR^®^ eco N 7300 by Covestro [23].

However, many reported applications of bio-based PU coatings are on hard substrates such as wood or metal and not on textile. This paper describes the synthesis, formulation, and application of bio-based waterborne PU on textiles. Bio-based polyol, diisocyanate and non-toxic catalyst were used as raw materials. The resulting PU was characterised, applied on polyester fabric and the coating properties were assessed.

## 2. Materials and Methods

### 2.1. Materials

Bismuth neodecanoate, triethylamine (TEA), dimethylol propionic acid (DMPA), diethylene triamine (DETA), methyl ethyl ketone (MEK) and isophorone diisocyanate (IPDI) were purchased from Sigma-Aldrich (Darmstadt, Germany). Respumit NF01, Edolan XTP and Edolan XCI were sampled by Tanatex Chemicals (Ede, The Netherlands). Priplast 3190 (polyester polyol) was sampled by Croda (Gouda, The Netherlands). Polyester fabric (105 g/m^2^) was purchased from Concordia Textiles (Waregem, Belgium).

### 2.2. Synthesis of Bio-Based PU Dispersion

**Figure 1:**
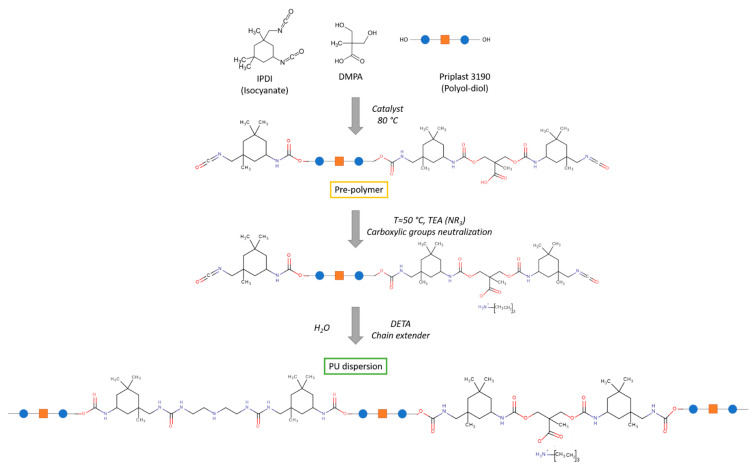
Bio polyurethane dispersion (PUD) synthesis scheme.

An isocyanate-terminated pre-polymer was prepared in a four-neck flask equipped with mechanical stirrer, nitrogen inlet, water condenser and thermometer. Total of 40 g Priplast 3190 and 3.54 g DMPA were weighed in the flask and 60 mL MEK was added. The solution was heated at 50 °C for 20 min under nitrogen to dissolve DMPA and afterwards temperature was increased to 80 °C. Subsequently 0.1 g of bismuth neodecanoate catalyst was added, followed by controlled addition of 15.84 g IPDI by using a dropping funnel. The reaction was monitored via FT-IR. After 4 h the reaction was cooled down to 50 °C and 1.78 g of TEA was added drop by drop to neutralise the pre-polymer. The pre-polymer was then dispersed in deionised water using an Ultra-Turrax disperser from IKA (Staufen, Germany), resulting in a milky white dispersion. MEK was removed by a rotary evaporator under reduced pressure at 40 °C and finally 1.7 g DETA (chain extender) was added. The solid content of the bio-based dispersion was calculated to be 40 wt%. The reaction is shown in Figure 1.

### 2.3. Fabric Coating

Total of 50 g of the developed bio-based PU dispersion and 2 g of Edolan XCI were mixed. About 0.3 g Respumit NF01 defoamer was added. The defoamer was added to remove air bubbles in the formulation which could have a negative impact on the resistance to hydrostatic pressure. Finally, Edolan XTP was added to increase the viscosity. The formulation was applied to a polyester fabric via knife over roll. The applied coating thickness was 100 µm. Three layers were applied and after applying each layer, the coating formulation was dried 1 min at 110 °C and cured for 2 min at 155 °C.

### 2.4. Characterisation

Fourier transform infrared spectra (in µ-ATR mode) were recorded using a Nicolet 6700 spectrometer from Thermo Fisher Scientific (Waltham, MA, USA). A spectral range from 500 cm^−1^ to 4000 cm^−1^ and a resolution of 4 cm^−1^ was used. The infrared analysis was used to characterise the bio-based PU dispersion. The surface morphology of the coating was visualised using a field emission gun scanning electron microscopy (FEG-SEM) (JSM 7600 F from Jeol Europe (Zaventem, Belgium)). To prevent charging, the specimens were sputtered with a palladium coating.

Thermogravimetric analysis (TGA) of the PU was performed to examine the thermal decomposition using a Q500 thermogravimetric analyser from TA Instruments (Asse, Belgium). All samples were conditioned at 23 °C and 50% relative humidity. Analyses were performed in air and nitrogen with a ramp rate of 10 °C/min from 30 °C to 600 °C. The thermograms were analysed using Universal Analysis Software (v5.5.24) from TA Instruments (Asse, Belgium).

Differential scanning calorimetry (DSC) was performed using TA Instruments Discovery DSC2500 from TA Instruments (Asse, Belgium). All samples were conditioned at 23 °C and 50% relative humidity. All samples were heated from 0 °C to 250 °C, cooled down from 250 °C to 0 °C and heated back from 0 °C to 250 °C. Analyses were performed with a heating and cooling rate of 10 °C/min.

The particle size and polydispersity index (PDI) of the bio-based dispersions were measured by a Nano-ZS90 Zeta-sizer from Malvern Instrument (Malvern, UK) at 25 °C. The sample was first diluted in deionised water prior measurement.

The solid content of the bio-based PUD was determined by drying 5.00 g of the dispersion at 110 °C until constant mass was obtained and weighing the mass of the solid residue. The solid content was calculated as the ratio of the mass of the solid residue to the mass of the dispersion initially (5.00 g).

The air permeability was assessed according to ISO 9237 using Textest FX 3300 apparatus from Artec Testnology (Kerkdriel, The Netherlands). The rate of a flow air passing perpendicularly through the coated fabric is measured at a pressure drop of 100 Pa across the fabric test area (20 cm^2^). Ten measurements were performed, and the average value is reported.

Elongation at break, stress at break and elasticity modulus were determined using Instron electronic fabric tension tester according to ISO 13934-1 on 40 µm PU films. The tension loading speed was 100 mm/min. The recovery after 5 stretch-strain cycles was assessed according to EN14704-1 using 40-µm PU films. The recovered elongation was measured after 1 min. The specimens were prepared according to the EN standard. The resistance to hydrostatic pressure was measured according to ISO 811 using Textest FX 800 apparatus. The coated fabric is subjected to a steadily increasing pressure of water on one face, under standard conditions, until penetration occurs in three places. The same test is repeated after washing and exposure to UV or high temperature and humidity to examine respectively wash fastness, QUV and hydrolysis resistance. Wash fastness was evaluated according to ISO 6330. Ten washing cycles at 40 °C were done in Wascator FOM 71 (type A). The detergent was ECE detergent (type 3). The samples were dried at room temperature after washing. Hydrolysis resistance was measured according to ISO 1419-C (tropical test), by exposing the coated fabric during 3 weeks to a temperature of 70 °C and a relative humidity of 95% and examining the resistance to hydrostatic pressure afterwards. Resistance towards ageing by means of heat, light and humidity (QUV test) was done according to ISO 4892. The details are given in Table 1. Generally, the total duration of the test was set at 500 h.

The antimicrobial properties were evaluated according to ISO 20743. The specimens were prepared according to the ISO standard. ISO 20743 test is used to quantitively measure the antimicrobial activity of textiles. Samples are inoculated by transfer of bacteria (*Staphyllococcus aureus* and *Klebsiella pneumoniae*, respectively Gram+ and Gram− bacteria) from a previously inoculated agar plate to the samples. This is done for both the coated polyester samples and the uncoated polyester samples, which are the control samples. For each, six samples are made. Immediately after the transfer, bacteria are extracted form 3 coated and 3 control samples and counted. The others (including control) are incubated at 37 °C for 24 h. After incubation, remaining bacteria are extracted and counted from 3 coated and 3 control samples. Based on these data, the antibacterial activity (A) is calculated as the difference in growth values on the control and the coated fabric.

The burning behaviour of the fabrics was assessed via ISO 15025 (surface and edge ignition). The specimens were prepared according to the ISO standard. This standard is used to determine the flame spread properties of vertically oriented materials, when subjected to a small defined flame. A defined flame from a specified burner is applied for 10 s to the surface or the bottom edge of textile specimens which are vertically oriented. Criteria such as afterflame time (flaming after removal of ignition source), afterglow (persistence of glowing combustion of material), formation of molten or flaming debris, hole formation and flame on edge are assessed.

The abrasion resistance was evaluated according to EN 530-2 using a Martindale wear and abrasion tester. The specimens were prepared according to the EN standard. The instrument was used in inverted mode, i.e., the specimen is placed on the abradant table instead of in the test piece holder and the abradant is mounted in the test piece holder. This provides an abraded area which allows measurement of resistance to hydrostatic pressure afterwards. The test was carried out for 500 cycles with F2 abradant (sandpaper) and an applied pressure of 9 kPa on the sample. After determination of the resistance to hydrostatic pressure, the mass loss was determined after 500 abrasion cycles.

## 3. Results and Discussion

### 3.1. Structural Characterisation of Bio-Based PUD

**Figure 2:**
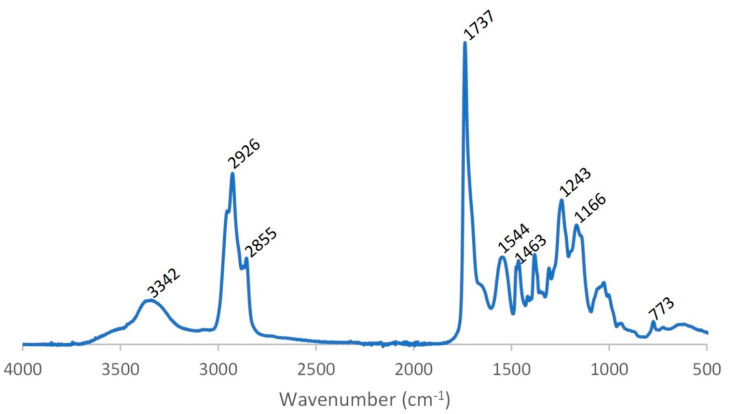
FT-IR spectrum of the developed bio-based PUD.

The bio-based PUD was characterised with FT-IR. Figure 2 demonstrates the FT-IR spectrum of the bio-based PUD. The absence of the asymmetrical N=C=O stretch between 2250 and 2285 indicates no presence of remaining isocyanates, indicating the PUD synthesis is completed. Some characteristic bands appeared in the FT-IR spectrum. Table 2 gives an overview of the different bands and the corresponding groups. The formation of urethane bands is mainly identified by the absence of OH groups and the appearance of NH bands.

The absorption band at 3342 cm^−1^ corresponds to NH stretching, while the band at 1737 cm^−1^ is attributed to C=O stretching. The peaks at 2855 cm^−1^ and 2926 cm^−1^ are associated with −CH2 stretching, while other modes of −CH2 vibrations are identified by the band at 1463 cm^−1^. The band at 1544 cm^−1^ is typically for N-H bending and C-N stretching in polyurethanes. The sharp peak at 1243 cm^−1^ can be attributed to C-O-C urethane vibrations, while the peak at 1166 cm^−1^ is assigned to C-O-C vibrations of the ester groups. The small peak at 773 cm^−1^ originates from COO urethane vibrations [24,25,26,27].

### 3.2. Dispersion and Coating Characteristics

**Figure 3:**
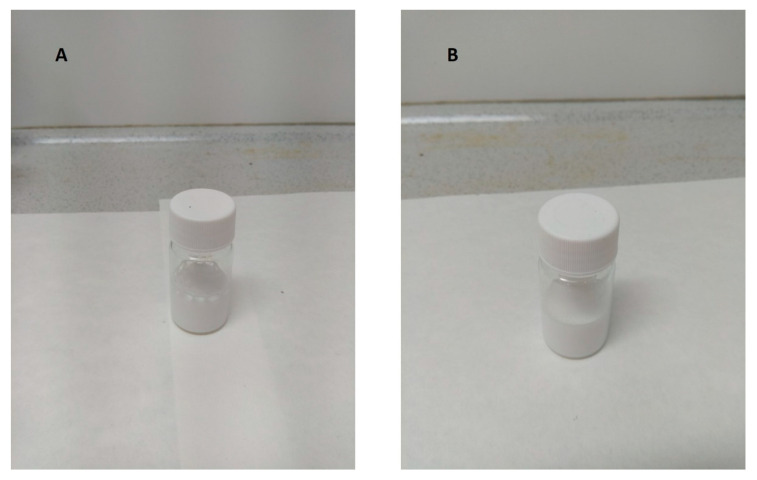
Bio-based PUD initially (**A**) and after storing 6 months at ambient temperature (**B**).

The average particle size of the developed bio-based PU dispersion was 98 nm and the polydispersity index (PDI) amounted to 0.150, which indicates a small particle size distribution. The solid content of the dispersion was 38.9%. The reported numbers of the bio-based PUD are in range with the particle size, PDI and solid content of commercial water-based PUD. The particle size of the evaluated commercial water-based PUD varied between 50 and 231 nm and the PDI varied between 0.016 and 0.269. The solid content of commercial water-based PU is in the range of 40–60%. The developed dispersion was monitored for 6 months at ambient temperature for any solid sedimentation or settling to assess shelf life stability (Figure 3). The percent of dispersed solids was measured in the stored PUD by decantation of the PUD after six months and measuring the dry solid content. After 6 months the solid dispersed content was 38.7%. Compared to the original solid content of 38.9%, this indicates a very low settlement of PU particles in the dispersion and a high stability of the PUD.

**Figure 4:**
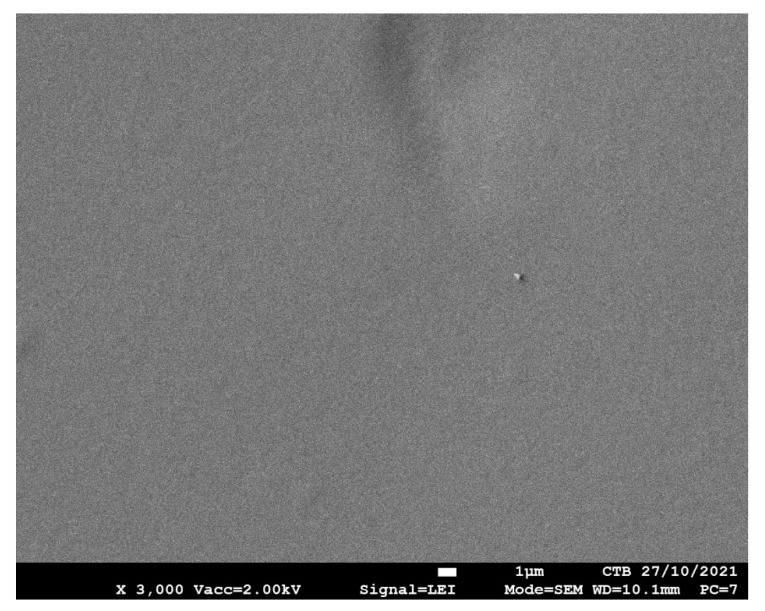
SEM image of bio-based PU coating.

The SEM image of the bio-based PU coating is shown in Figure 4. The PU coatings have a smooth and homogeneous surface morphology. No cracks or other defects due to poor wetting or air inclusion were observed.

### 3.3. Mechanical Characteristics

**Figure 5:**
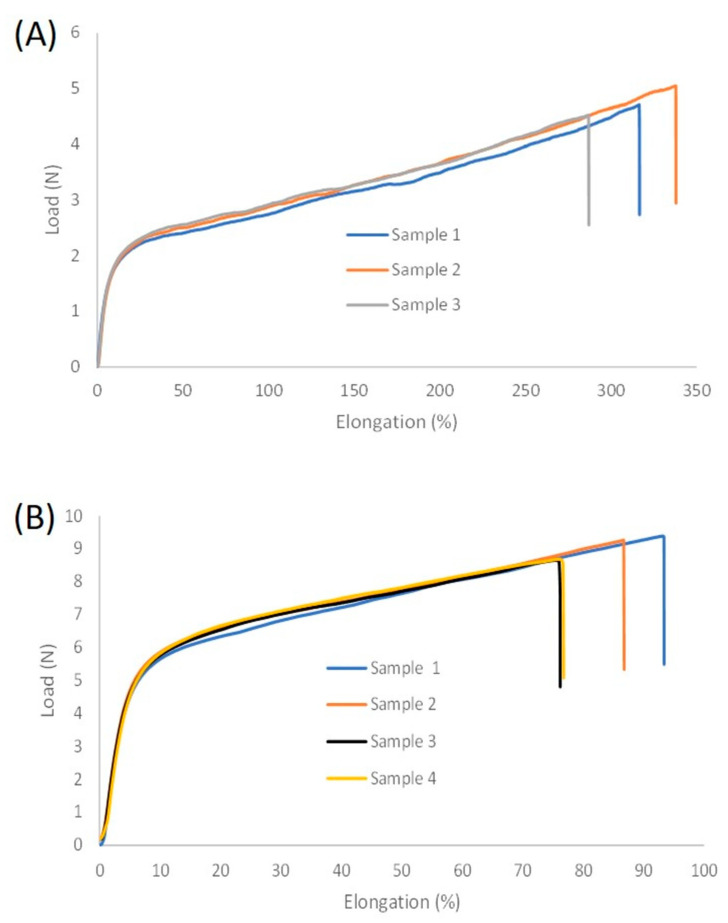
Tensile curves of bio-based PUD film without crosslinker (**A**) and with crosslinker (**B**). 3 tensile tests were performed on bio-based PUD film without crosslinker and 4 tests were performed on bio-based PUD film with crosslinker.

Elongation of the coating was assessed according to ISO 13934-1 on 40 µm PU films of the bio-based PUD. The tensile curves are shown in Figure 5. The effect of adding extra polyisocyanate crosslinker (Edolan XCI) on the elongation was also studied (Table 3). Average values of at least three measurements were reported. The elongation of the bio-based PUD was higher than 300%, which makes it possible to apply the coating also on knitted fabrics via transfer coatings. Indeed, knitted fabrics have higher elasticity and flexibility compared to woven fabrics, causing coatings with low elongations to tear on knitted fabrics and not on woven fabrics. The bio-based PU films with extra polyisocyanate crosslinker exhibited a much smaller elongation. Addition of cross-linker will result in a more rigid network and lower chain mobility, due to reaction of the crosslinker with remaining functional groups in the backbone of the PU, causing a decrease in elongation, flexibility and elasticity. However, the crosslinker increased the stress at break and the elasticity modulus of the bio-based film. Crosslinking reinforces the film resulting in higher tensile stress at break. However, further increase of crosslinking might result in a decrease of stress at break caused by flexibility loss and less force is needed to break the sample when exposed to stress [28]. Elasticity modulus is calculated as the ratio of stress to strain along the initial slope of the stress–strain curve. Since addition of crosslinker results in a decrease of chain mobility, the elasticity modulus of the crosslinked film is, as expected, higher compared to the bio-based PU film without crosslinker. Generally, the elasticity modulus of the bio-based PU is low, indicating that it is a soft material making it applicable for textile coatings since these need to be stretchable and flexible. The 100% modulus of the bio-based PU film is 1.4 MPa. The 100% modulus of the crosslinked film could not be calculated since the elongation at break was lower than 100%. The recovered elongation of the bio-based PUD after five stress–strain cycles was excellent: 97% and 99.2% for the non-crosslinked and the crosslinked PU, respectively. Small increase of crosslinking density resulted in a slight increase of recovered elongation. The air permeability of the polyester fabric coated with the bio-based PUD was lower than 1.00 L/(m^2^ s).

### 3.4. Thermal Behavior

**Figure 6:**
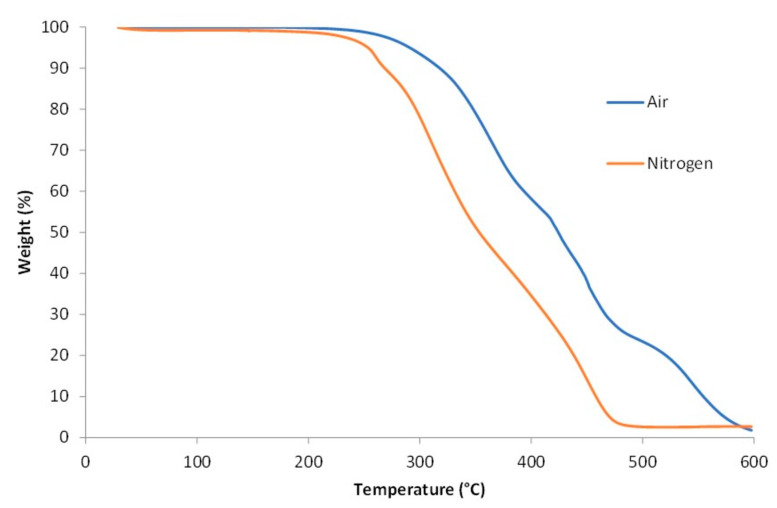
TGA curves and the corresponding derivative curves from bio-based PUD in air (above) and nitrogen (below) atmosphere.

The thermal degradation of bio-based PU coating was analysed via TGA up to 600 °C in air and nitrogen atmosphere. Almost no mass residue was observed at 600 °C indicating that the bio-based PU was completely degraded, and no char was formed. In both air and nitrogen atmosphere the degradation started above 200 °C (Figure 6). However, in nitrogen atmosphere the bio-based PU degraded faster. This was in agreement with the results reported by Gu et al. and is attributed to the lower thermal conductivity of nitrogen compared to air [29]. The first degradation step is associated with the dissociation of the hard urethane segments resulting in isocyanate, alcohol, CO_2_, primary and secondary amines and olefins. The second degradation step is attributed to the dissociation of ester groups and linear hydrocarbon chains, while the degradations in the third step are due to C=C cleavage [30,31,32]. Degradation between 500 °C and 600 °C in air can be assigned to oxidative degradation of the structure.

**Figure 7:**
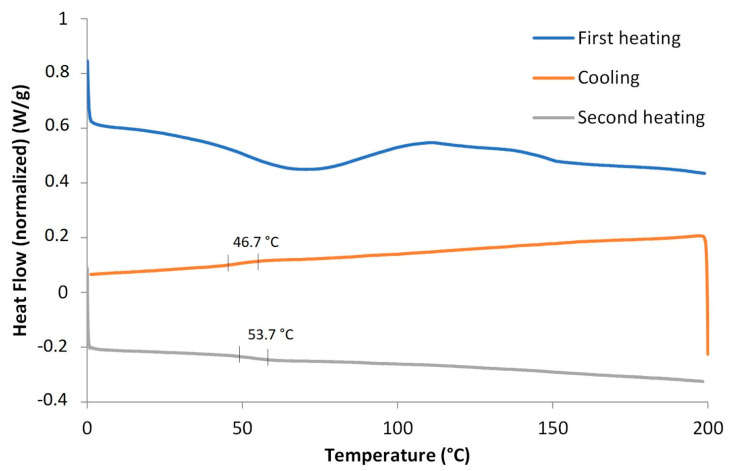
DSC analysis of bio-based PUD during subsequent heating (blue), crystallisation (orange) and heating (grey) in a temperature range of 0 °C–200 °C. The first heating is shown in green.

Differential scanning calorimetry (DSC) analysis revealed that the Tg (glass transition temperature) of the coating amounted to 53.7 °C during the second heating step (Figure 7). The transition does not occur suddenly at one unique temperature but rather over a small range of temperatures. The temperature in the middle of the sloped region was taken as the Tg. The coating showed no melting behaviour indicating that the coating was a thermoset and not thermoplastic and therefore could not be welded contrary to solvent-based thermoplastic PU coatings.

### 3.5. Performance of the Bio-Based PUD Coating

The resistance to hydrostatic pressure of bio-based PUD-coated polyester fabric initially and after QUV ageing, hydrolysis or washing was measured. The maximum resistance to hydrostatic pressure that could be measured with the equipment was 1000 mbar. The results are listed in Table 4. The coated fabrics showed excellent resistance to hydrostatic pressure (1000 mbar) initially. After exposing to 70 °C and 95% relative humidity for 3 weeks (hydrolysis test), the resistance to hydrostatic pressure was not changed and no degradation of the coated polyester fabric was observed. After 500 h ageing (QUV test) light yellow discoloration of the bio-based PU coating and a minor decrease of the resistance to hydrostatic pressure from 1000 mbar to 970 mbar were observed. The results demonstrate that the developed bio-based PU coating has excellent resistance to hydrolysis and QUV ageing. The resistance to hydrolysis could be ascribed to the hydrophobic soft segment of the PU. The wash fastness of the coating was evaluated, by measuring the resistance to hydrostatic pressure after ten washing cycles at 40 °C. After washing the coating showed no defects and still exhibited excellent resistance to hydrostatic pressure. The test results indicate that the developed bio-based PUD has an excellent durability and high performance.

The abrasion resistance of the polyester fabric coated with the developed bio-based PUD was assessed via Martindale. The mass loss after 500 abrasion cycles with sandpaper amounted to 22 mg, which was less than 1% of the total mass of the coated textile (the weight and surface of the test area of the coated textile was 2.769 g and 154 cm^2^, respectively) or less than 2% of the mass of the bio-based PUD coating in the test area, which was calculated to be 1.152 g based on the mass of the uncoated polyester fabric (105 g/m^2^). The resistance to hydrostatic pressure after 500 abrasion cycles with sandpaper decreased significantly to 143 mbar, but was still above the threshold of 100 mbar, which is set for many technical textile applications.

The antimicrobial properties were assessed by determining the antibacterial activity (A) of the coated samples against *Staphylococcus aureus* and *Klebsiella pneumoniae* (ISO 20743). The antibacterial activity is significant if 2 ≤ A < 3 or strong if A ≥ 3. DETA was used as chain extender in the synthesis of the bio-based PUD, causing the PUD to possess tertiary amine groups, which is not the case when other chain extenders as butanediol or ethylene diamine would have been used. Since tertiary amine groups are known for their antimicrobial activity, the antibacterial properties of the resulting bio-based PUD were examined in comparison with uncoated polyester fabric [33]. The results are shown in Table 5. Polyester fabric coated with the developed bio-based PUD showed less bacterial growth compared with uncoated polyester fabric. However, the antibacterial activity cannot be considered to be significant since the values are below 2. PU is known to be susceptible to bacterial colonisation unless antibacterial additives are added [34]. Increasing the content of DETA would improve the antibacterial properties because of the higher amount of tertiary amine groups, but this results in harder and less flexible coatings due to an increase of the hard segments in the PU backbone.

Polyester fabric and bio-based PUD coated polyester fabric were tested according to ISO 15025 (surface and edge ignition). Polyester fabric burned heavily and showed no flame retarding properties as expected. In case of surface ignition, the samples were burned completely after 4 s. Flaming and molten debris were also noticed, igniting the paper below the sample. After edge ignition it took a bit longer for the polyester samples to be burned completely. Again, flaming and molten debris were noticed, leading to the ignition of paper, which was put below the sample (Table 6). Polyester coated with the developed bio-based PUD was subjected to the same test, however the coated samples showed no ignition, no afterflame time or any afterglow. Furthermore, contrary to uncoated polyester the samples did not burn and did not produce flaming or molten debris. It could be concluded that the developed bio-based PUD coating had flame retardant properties, probably due to the presence of nitrogen in the backbone as a result of incorporation of DETA as a chain extender.

## 4. Conclusions

In summary, bio-based PUD coatings, specifically designed for textile coatings, were synthesised and applied via knife coating on polyester fabric. FT-IR analysis was performed to characterise the bio-based coating. TGA analysis showed that degradation started above 200 °C. The Tg of the coating was 53.7 °C, but the PUD has no melting point. The elongation of the bio-based PUD was higher than 300%. Generally, the elongation of commercial bio-based PU coatings (both solvent-based and water-based) ranges between 300% and 1200%. Addition of extra isocyanate crosslinker to the PUD decreased the elongation substantially. The developed bio-based PU coating exhibited excellent hydrostatic pressure, QUV ageing resistance, hydrolysis resistance and wash fastness at 40 °C but moderate abrasion resistance. In comparison, solvent (DMF)-based PU coatings show better wash fastness at 60 °C and 90 °C and are weldable due to their thermoplastic properties. However synthetic polyester PU (both water-based and solvent-based) are sensitive to hydrolysis and additives need to be added to improve the hydrolysis resistance, contrary to the bio-based PUD. Synthetic water-based crosslinked PU coatings also show better wash fastness at 60 °C and in some cases even at 90 °C, but only in case of polycarbonate PU. Due to additional crosslinking (needed to obtain good wash fastness properties) most water-based PU coatings are not thermoplastic. Polyester fabric coated with the bio-based PUD passed ISO 15025 fire test without the need of FR additives in the coating formulation. Polyester fabric coated with the developed bio-based PUD showed less bacterial growth compared with uncoated polyester fabric. However, the antibacterial activity cannot be significant and therefore antibacterial agents still need to be added to have a significant antibacterial effect.

## Figures and Tables

**Table 1:** Experimental details of QUV test.

Light Source	UVA 340
Mode of exposure	Alternate light/condensation: 0.76 W/m^2^ at 340 nm
Temperature	60 °C
Duration of ageing	4 h
Condensation	50 °C
Duration of ageing	4 h

**Table 2:** Overview of functional groups of bio-based PUD detected by FT-IR.

Bio-Based PUD	
Wavenumber (cm^−1^)	Corresponding Group
773	COO urethane (deformation vibration)
1166	C-O-C ester (elongation vibration)
1243	C-O-C urethane (elongation vibration)
1463	CH (deformation vibration)
1544	N-H and C-N amide
1737	C=O urethane and ester (elongation vibration)
2855	CH (elongation vibration)
2926	CH (elongation vibration)
3342	NH (elongation vibration)

**Table 3:** Mechanical properties of (crosslinked) bio-based polyurethane (PU) films.

	Elongation (%)	Stress at Break (MPa)	Elasticity Modulus (MPa)
Bio-based PUD	314 ± 25	2.6 ± 0.1	0.20 ± 0.01
Bio-based PUD + 4% Edolan XCI	83 ± 8	5.0 ± 0.2	0.77 ± 0.02

**Table 4:** Performance of bio-based PU-coated polyester fabric.

Resistance to Hydrostatic Pressure (mbar)			
Initial	QUV Ageing	10 Washing Cycles (40 °C)	Hydrolysis
≥1000	970	≥1000	≥1000

**Table 5:** Antibacterial activity against *Staphylococcus aureus* and *Klebsiella pneumoniae*.

Antibacterial Activity	
*Staphylococcus aureus*	*Klebsiella pneumoniae*
1.2	1.1

**Table 6:** Overview of ISO 15025 results.

Surface Ignition						
Sample	Afterflame (s)	Afterglow (s)	Flaming/molten debris	Flame on Edge	Hole on Edge	Hole Formed
Polyester	4	0	yes	yes	yes	yes
Coated polyester	0	0	no	no	no	yes
**Edge Ignition**						
Sample	Afterflame (s)	Afterglow (s)	Flaming/Molten Debris		Flame on Edge	
Polyester	17	0	yes		yes	
Coated polyester	0	0	no		no	

## Data Availability

The data presented in this study are available on request from the corresponding author.
